# Efficient Heterogeneous Palladium Catalysts in Oxidative Cascade Reactions

**DOI:** 10.1021/acs.accounts.1c00122

**Published:** 2021-04-19

**Authors:** Man-Bo Li, Jan-E. Bäckvall

**Affiliations:** † Institute of Physical Science and Information Technology, Anhui University, Hefei, Anhui 230601, P.R. China; ‡ Hefei National Laboratory for Physical Sciences at the Microscale, Hefei, Anhui 230601, P.R. China; § Department of Organic Chemistry, Arrhenius Laboratory, Stockholm University, SE-10691 Stockholm, Sweden; ∥ Department of Natural Sciences, Mid Sweden University, SE-85170 Sundsvall, Sweden

## Abstract

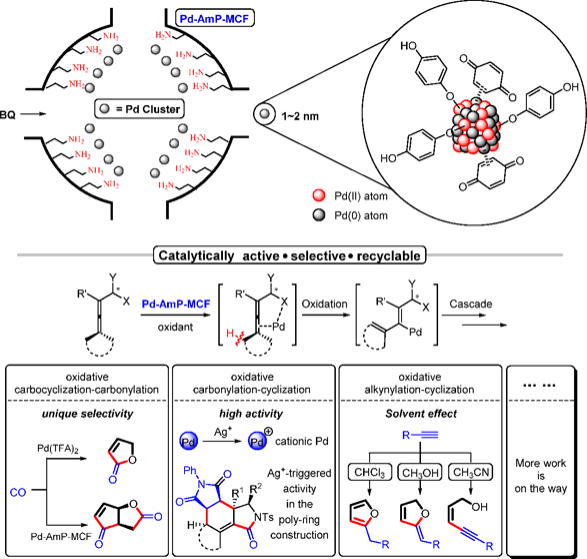

Palladium-catalyzed oxidations involving cascade processes provide a versatile platform for streamlined conversion of simple feedstocks into functional molecules with high atom and step economy. However, the achievement of high palladium efficiency and selectivity in Pd-catalyzed oxidative cascade reactions is still challenging in many cases, as a result of the aggregation of active palladium species to Pd black and the possible side reactions during each bond-forming step. The two current solutions for addressing these issues are either to utilize oxidant-stable ligands or to use electron transfer mediators (ETMs). The former solution, which includes the use of amines, pyridines, sulfoxides, and carbene derivatives, inhibits aggregation of Pd^0^ during the catalytic cycle, while the latter solution facilitates reoxidation of Pd^0^ to Pd^II^ to improve the activity and selectivity. Following our long-standing interest in Pd-catalyzed oxidations, very recently we developed heterogeneous catalysts to resolve the issues mentioned above in oxidative cascade reactions. The heterogeneous palladium catalysts (Pd-AmP-MCF or Pd-AmP-CNC) comprise palladium nanoclusters (1–2 nm) immobilized on amino-functionalized siliceous mesocellular foam (MCF) or on crystalline nanocellulose (CNC), exhibiting high activity, selectivity as well as excellent recycling ability.

In this Account, we will discuss the synthesis and characterizations of the heterogeneous palladium catalysts, as well as their catalytic behaviors, and the mechanisms involved in their reactions. An important aspect of these catalysts in oxidation reactions is the generation of active Pd(II) species within the heterogeneous phase. Typical oxidative cascade reactions of our recent research on this topic include oxidative carbocyclization-carbonylation, oxidative carbocyclization-borylation, oxidative alkynylation-cyclization, oxidative carbonylation-cyclization, and oxidative carbocyclization-alkynylation. These reactions provide access to important compounds attractive in medicinal chemistry and functional materials, such as γ-lactone/γ-lactam-based poly rings, cyclobutenols, highly substituted furans, and oxaboroles. During these processes, the heterogeneous catalysts exhibited much higher turnover numbers (TONs) than their homogeneous counterparts (e.g., Pd(OAc)_2_) as well as unique selectivity that cannot be achieved by homogeneous palladium catalysts. The origin of the high efficiency and unique selectivity of the heterogeneous catalysts was also investigated. Asymmetric syntheses for the construction of optically pure compounds were realized based on the excellent selectivity in these heterogeneous processes. Kinetic studies revealed that the rate and yield of the reactions were essentially maintained during recycling, which demonstrates that Pd-AmP-MCF and Pd-AmP-CNC are robust and highly active in these oxidative cascade reactions. In addition, inductively coupled plasma optical emisson spectroscopy (ICP-OES) analysis and hot filtration test suggest that these processes most likely proceed via a heterogeneous pathway.

Recent progress in our group has shown that the activity of Pd-AmP-MCF and Pd-AmP-CNC could be improved even further by the addition of Ag^+^ to generate cationic Pd(II). Furthermore, intriguing solvent effects were observed in a Pd-AmP-MCF-catalyzed oxidative cascade process, and solvent-controlled chemoselective transformations were developed based on this property of the catalyst. The heterogeneous strategy of this Account provides solutions to palladium deactivation and selectivity issues in Pd(II)-catalyzed oxidative cascade reactions and enables efficient catalyst recycling, which will open up new opportunities in oxidative cascade reactions.

## Key References

- LiM.-B.; IngeA. K.; PosevinsD.; GustafsonK. P. J.; QiuY.; BäckvallJ.-E.Chemodivergent and Diastereoselective Synthesis of γ-Lactones and γ-Lactams: A Heterogeneous Palladium-Catalyzed Oxidative Tandem Process. J. Am. Chem. Soc.2018, 140, 14604–146083035839910.1021/jacs.8b09562.^1^*Unique selectivity in oxidative carbonylations of enallenols was observed for the first time by using Pd-AmP-MCF as the heterogeneous palladium catalyst.*
- LiM.-B.; PosevinsD.; GeoffroyA.; ZhuC.; BäckvallJ.-E.Efficient Heterogeneous Palladium-Catalyzed Oxidative Cascade Reactions of Enallenols to Furan and Oxaborole Derivatives. Angew. Chem., Int. Ed.2020, 59, 1992–199610.1002/anie.20191146231729824.^2^*Solvent-controlled chemoselectivity in Pd-AmP-MCF-catalyzed oxidative alkynylations was developed for the construction of different cyclized compounds.*
- LiM.-B.; YangY.; RafiA.; OschmannM.; GrapeE. S.; IngeA. K.; CórdovaA.; BäckvallJ.-E.Silver-Triggered Activity of a Heterogeneous Palladium Catalyst in Oxidative Carbonylation Reactions. Angew. Chem., Int. Ed.2020, 59, 10391–1039510.1002/anie.202001809PMC746317432091647.^3^*AgOTf is able to remove Cl*^*-*^*on the surface of Pd-AmP-MCF, creating cationic Pd, which is highly active in oxidative carbonylation cascade reactions.*
- LiM.-B.; YangJ.; YangY.; XuG.-Y.; LuoG.; YangJ.; BäckvallJ.-E.Amino-Supported Solid Palladium Catalyst for Chemo- and Stereoselective Domino Reactions. Angew. Chem., Int. Ed.2021, 60, 670–67410.1002/anie.202011708PMC783973032969105.^4^*The amino linker/ligand on Pd-AmP-MCF protects Pd from aggregation and regulates catalytic selectivity, resulting in high efficiency of the heterogeneous palladium catalyst in oxidative carbocyclization-alkynylation reactions.*

## 1 Introduction

**Scheme 1:**
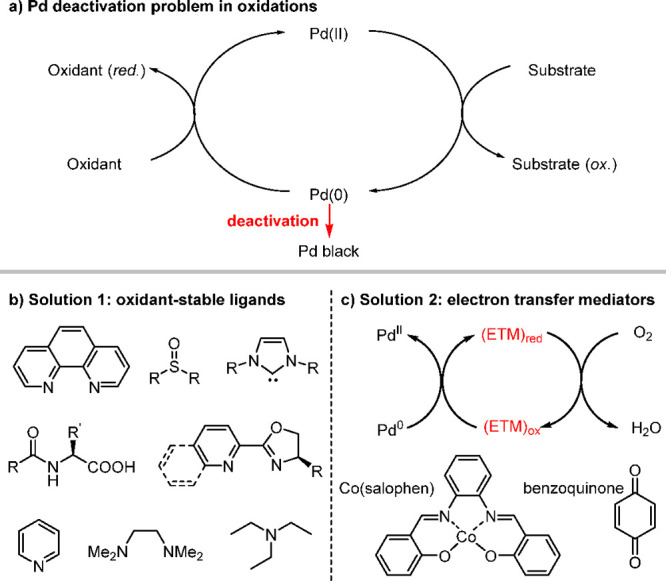
Palladium Deactivation Problem in Oxidative Cascade Reactions (a) and the Two Current Solutions (b and c)

Palladium-catalyzed oxidations provide the basis for streamlined conversion of simple feedstocks into valuable products.^5−7^ The interest in this research field originates from the middle of the 1950s by the discovery of the Wacker process,^8^ which was responsible for an annual production of over one billion pounds of acetaldehyde from ethylene at one point. The developed methodologies of Pd-catalyzed oxidations not only contribute to solve the poor selectivity problem in oxidation reactions, but also stimulate the progress of synthetic organic chemistry. Up to now, a wide range of oxidations have been realized based on homogeneous palladium catalysts, and illustrative examples include alkene and diene functionalizations,^9,10^ alcohol oxidations,^11^ and C–H activations.^12,13^ In particular, these oxidative processes with subsequent cascade reactions enable diverse functionalizations of organic molecules, which provide a versatile platform for the construction of bioactive compounds and other functional molecules.^14,15^ In spite of the significant progress in Pd-catalyzed oxidative cascade reactions, the achievement of high palladium efficiency is still challenging in many cases.^16,17^ One of the major reasons is the aggregation of active palladium species to Pd black under homogeneous conditions (Scheme 1a), which results in the deactivation of the palladium catalyst. Additionally, although the oxidative cascade reactions lead to target products with high atom and step economy, the control of selectivity during these processes is difficult, because of the diverse side pathway during each bond-forming step. Effective catalyst systems that address the issues of palladium deactivation and selectivity control in oxidative cascade reactions is always in high demand. In this context, considerable efforts have been made by different groups, and two efficient solutions have been developed to improve the activity and selectivity of homogeneous palladium catalysts. One of them is the utilization of oxidant-stable ligands such as sulfoxides, carbenes, and amines, which were developed by the Yu, White, Stahl, and other research groups.^18−20^ These ancillary ligands improve the efficiency of the palladium catalyst by means of inhibiting aggregation of the Pd^0^ intermediate during the catalytic cycle (Scheme 1b). The other solution is the application of electron transfer mediators (ETMs) such as a metal macrocycle (e.g., Co(salophen), FePc) together with benzoquinone (BQ),^21,22^ which facilitates reoxidation of Pd^0^ to active Pd^II^, via low-energy electron transfer, thus improving the efficiency of the palladium catalyst (Scheme 1c).

The use of oxidant-stable ligands or electron transfer mediators in homogeneous Pd-catalyzed oxidative cascade reactions has played an important role for improving the catalytic turnover and selectivity. Meanwhile, it has been noticed that, among some of these cases, the real active catalyst species were in situ generated palladium clusters. In 1995, the Hiemstra group reported that the Pd(OAc)_2_/DMSO catalytic system efficiently catalyzed the oxidative cyclization of *N*-hydroxymethylamine.^23^ Based on transmission electron microscopy (TEM), it was shown that Pd clusters were generated during the reaction and they could be isolated and reused without loss of catalytic activity. Results from the Sheldon group demonstrated that Pd nanoclusters, formed in situ during the reaction, were catalytically active in the alcohol oxidation.^24^ Stahl and co-workers found that in the oxidation of cyclohexanones and cyclohexenones to phenols with the Pd(TFA)_2_/2-dimethylaminopyridine catalytic system, the initial Pd^II^ catalyst evolved to Pd clusters immediately and catalyzed the formation of phenols.^25^ Additionally, Pd clusters exhibited distinct selectivity compared to monomeric palladium catalysts. Control experiments showed that monomeric palladium catalysts, disfavoring the formation of Pd clusters, catalyzed oxidation of cyclohexanone to cyclohexenone without subsequent oxidation to phenol. It is noteworthy that the continual growth of Pd clusters to nanoparticles (>100 nm) leads to loss of activity in these oxidation reactions. These reports together suggest that Pd clusters with small size would overcome the mass-transfer issue, generally associated with heterogeneous catalysts, and provide high activity in oxidative cascade reactions. Moreover, these clusters can also alter the catalytic selectivity compared with the monomeric palladium catalysts. Additionally, the easy removal of Pd clusters from the reaction mixture enables catalyst recycling, which is highly beneficial concerning practical applications in the fine chemical and pharmaceutical industry. However, unlike the wide application of Pd nanoparticles in Pd^0^-catalyzed cross-coupling reactions,^26,27^ heterogeneous nanopalladium-catalyzed oxidative cascade processes are still quite limited.^28,29^ A reasonable explanation might be that the relatively harsh oxidative reaction conditions cause problems to control the activity and selectivity. Also, the oxidative conditions may lead to Pd leaching problems, which make it difficult to identify the real active species and catalytic sites.

Our group has a long-standing interest in Pd-catalyzed oxidations,^5,21a,30^ and recently we have been involved in the oxidative cascade transformations of allenes.^31,32^ In the majority of cases, relatively high palladium loading (≥5 mol %) was needed for satisfactory conversion, and the control of selectivity was also challenging in some cases. Meanwhile, we designed and prepared a palladium catalyst which comprises small palladium clusters immobilized in amino-functionalized siliceous mesocellular foam (Pd-AmP-MCF).^33^ This heterogeneous Pd catalyst has been applied in various organic transformations, such as selective hydrogenation,^34^ cycloisomerization,^35^ and water splitting.^36^ Not only has Pd-AmP-MCF been shown to display high activity, it has also proven to be recyclable during these reactions. Particularly, the combination of Pd-AmP-MCF with an enzyme (*Candida antarctica* lipase B, CALB) has been demonstrated to be highly efficient in dynamic kinetic resolution of primary amines,^37^ in which the nanopalladium promotes racemization of amines. Inspired by these results and the previous reports on Pd cluster-catalyzed oxidations, we envisioned that Pd-AmP-MCF would be catalytically efficient in oxidative cascade transformation of allenes for two reasons: (1) the palladium clusters with ultrasmall size would circumvent the mass-transfer issue; (2) the amino linker and porous support would hold the palladium clusters firmly, avoiding Pd aggregation or leaching. After about 3 years of work, we have successfully developed a series of the heterogeneous palladium-catalyzed oxidative cascade reactions of allene compounds by using BQ as the terminal oxidant.^1−4,38^ During these transformations, Pd-AmP-MCF and its analogue Pd-AmP-CNC^3^ exhibited much higher turnover numbers (TONs) than their homogeneous counterparts (e.g., Pd(OAc)_2_). In some cases, the heterogeneous catalysts showed unique selectivity for the construction of complex molecules that cannot be achieved by homogeneous palladium catalysts. In addition, Pd-AmP-MCF and Pd-AmP-CNC were robust under the oxidative reaction conditions and no Pd leaching or aggregation was observed. It was demonstrated that the rate and yield of the oxidative cascade reactions were essentially maintained during recycling. In this Account, we discuss our recent developments on the oxidative cascade methodology of allenes to important compounds attractive in medicinal chemistry and functional materials, with a focus on the efficient heterogeneous palladium catalysts. The high activity, unique selectivity, and solvent effects in these transformations are summarized, and finally an introspective outlook is presented concerning heterogeneous palladium-catalyzed oxidations.

## 2 Synthesis of the Heterogeneous Palladium Catalysts and the Initial Attempts in Oxidative Cascade Reaction

### 2.1 Synthesis of Pd-AmP-MCF and Pd-AmP-CNC

**Figure 1:**
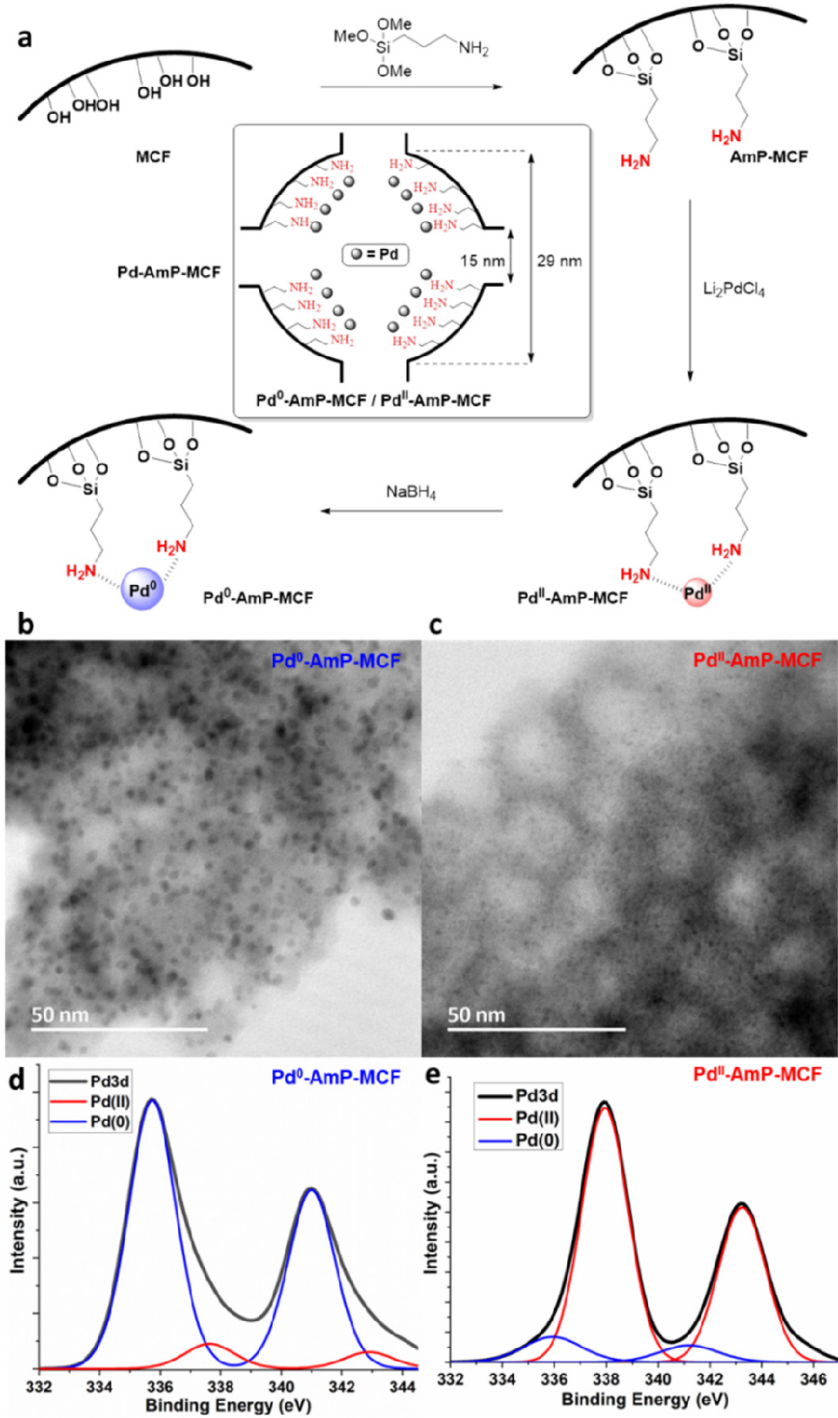
Synthetic strategy of Pd-AmP-MCF (a). TEM and XPS of Pd^0^-AmP-MCF (b and d) and Pd^II^-AmP-MCF (c and e).

Siliceous mesocellular foam (MCF) was used as the solid support, which was functionalized by 3-aminopropyltrimethoxysilane to introduce an amino group. Aminopropyl-functionalized MCF (AmP-MCF) was then impregnated with Li_2_PdCl_4_ to furnish the Pd^II^-AmP-MCF, which was reduced by NaBH_4_ to give Pd^0^-AmP-MCF. Both Pd^II^-AmP-MCF and Pd^0^-AmP-MCF were used in the oxidative cascade reactions discussed in this Account, and they are collectively called Pd-AmP-MCF. The pore and window sizes of Pd^II^-AmP-MCF and Pd^0^-AmP-MCF were measured and found to be essentially the same as those in nonfunctionalized MCF: 29 and 15 nm (Figure 1a). TEM images showed the average cluster size of Pd^0^-AmP-MCF to be 1–2 nm (Figure 1b), while the unreduced catalyst Pd^II^-AmP-MCF bears much smaller (0.4 nm) palladium clusters (Figure 1c). Results from X-ray photoelectron spectroscopy (XPS) indicate that the surface of the palladium clusters in Pd^0^-AmP-MCF (Figure 1d) and Pd^II^-AmP-MCF (Figure 1e) are dominated by Pd(0) and Pd(II), respectively. By using ecofriendly crystalline nanocellulose (CNC) in place of MCF as the support, we also synthesized Pd^0^-AmP-CNC and Pd^II^-AmP-CNC catalysts^3^ with the same procedure as in Figure 1a.

### 2.2 The Initial Use of Pd-AmP-MCF in an Oxidative Cascade Reaction

**Figure 2:**
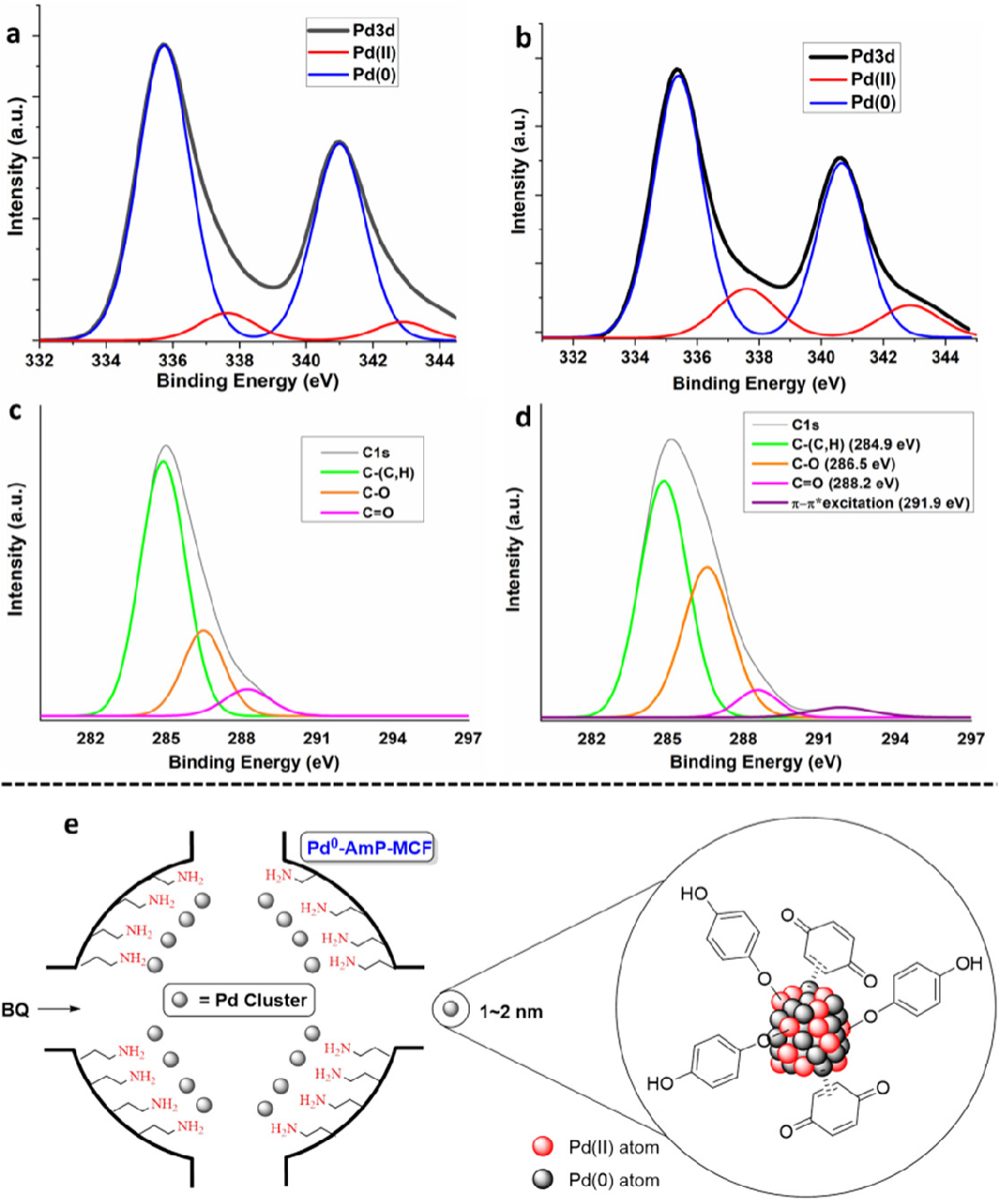
XPS spectra of Pd^0^-AmP-MCF before (a and c) and after (b and d) treatment by BQ, and the illustration of the activated catalyst (e, we are not sure about the real status of the catalyst during the reaction; the cartoon of the Pd cluster is based on our XPS analysis, which showed the coexistence of Pd(0) and Pd(II)).

One of the major concerns with the use of Pd-AmP-MCF as the catalyst for oxidative cascade reactions was whether the oxidant could interact with the surface of the palladium cluster to generate and maintain active Pd(II) for the catalytic cycle. With this question in mind, we treated Pd^0^-AmP-MCF with BQ and then characterized it by XPS. The deconvoluted Pd 3d XPS spectrum showed that there was a significant increase of the Pd(II)/Pd(0) ratio from about 5/95 to 25/75 on treatment of Pd^0^-AmP-MCF with BQ (Figure 2a and b).^38^ Additionally, compared to the untreated Pd^0^-AmP-MCF, the deconvoluted C 1s XPS spectrum of Pd^0^-AmP-MCF after treatment by BQ showed increased proportions of C–(C,H), C–O, and C=O (Figure 2c and d). Particularly, a new peak identified to be π–π* excitation was observed after the BQ treatment.^38^ These results together indicate that the surface of the palladium clusters is partially oxidized by BQ and then BQ and the reduced hydroquinone (HQ) coordinate to Pd atoms on the surface. These generated Pd(II) atoms in the clusters could be the active sites required for oxidative cascade reactions (Figure 2e). The treatment of Pd^II^-AmP-MCF with BQ did not result in any obvious change of the Pd 3d XPS spectra, and Pd(II) was still dominant.

**Scheme 2:**
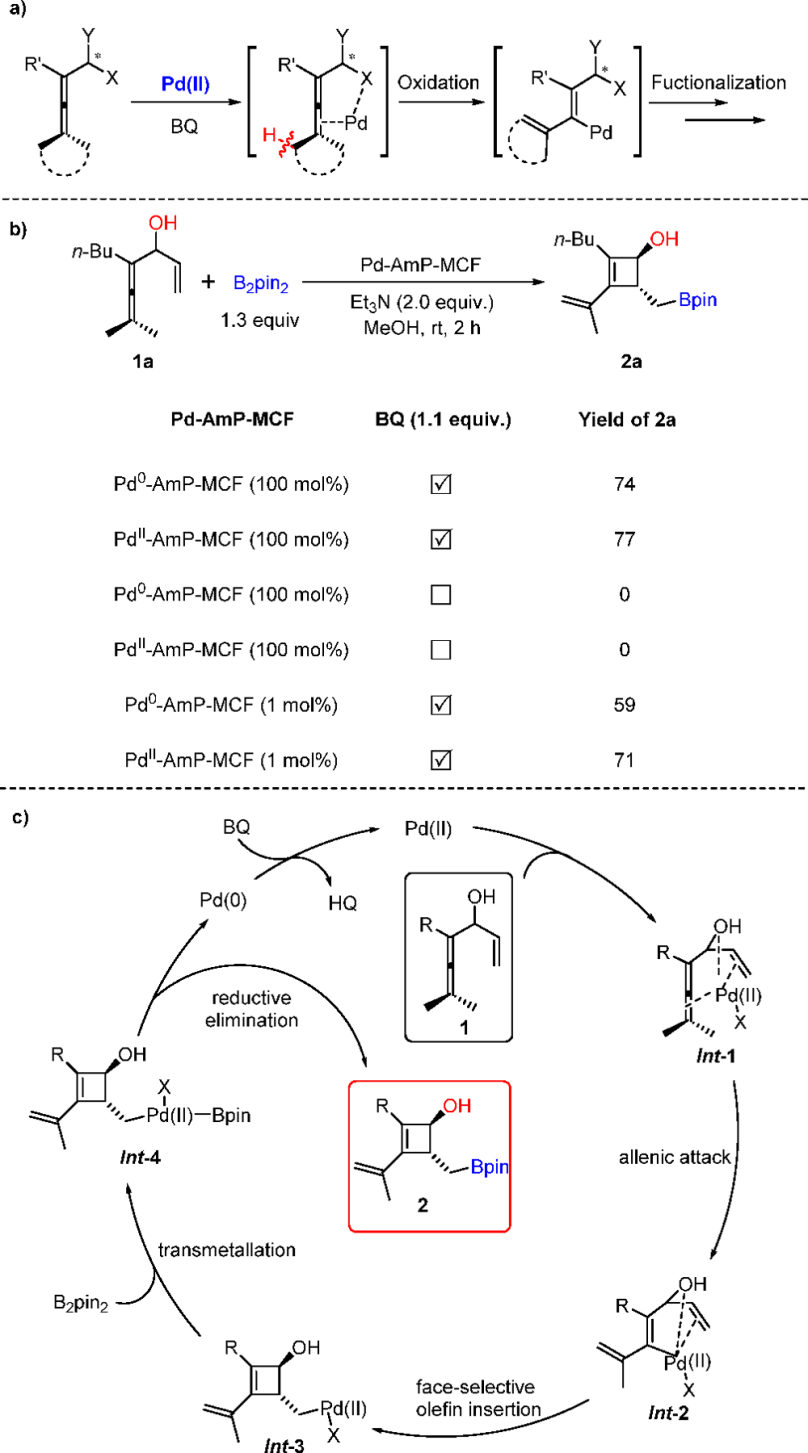
An Initial Attempt of Pd-AmP-MCF in an Oxidative Carbocyclization-Borylation Reaction

In the early 2010s, our group developed Pd-catalyzed oxidative functionalizations of allene compounds bearing an unsaturated moiety (Scheme 2a, X = olefin, alkyne, or allene).^31^ In these transformations, the catalysts were homogeneous palladium salts such as Pd(OAc)_2_ or Pd(TFA)_2_. To check the activity of the Pd-AmP-MCF prepared and discussed above, we designed an oxidative carbocyclization-borylation process (Scheme 2b).^38^ With enallenol **1a** and bis(pinacolato)diboron (B_2_pin_2_) as the reaction partners, stoichiometric experiments were initially tried. The results showed that both Pd^0^-AmP-MCF and Pd^II^-AmP-MCF were active with BQ as the additive. However, control experiments demonstrated that the absence of BQ completely shut down the reaction (Scheme 2b). The catalytic experiments showed that both Pd^0^-AmP-MCF and Pd^II^-AmP-MCF catalyzed the reaction in the presence of BQ as the oxidant, giving the desired product cyclobutenol **2a** in 59 and 71% yield, respectively. These results demonstrate that the heterogeneous palladium catalyst Pd-AmP-MCF is able to trigger the oxidative cascade process and complete the catalytic cycle below (Scheme 2c). Meanwhile, BQ is essential in the reaction and plays an important role not only as an oxidant, but also as a ligand to promote the transformation.

**Scheme 3:**
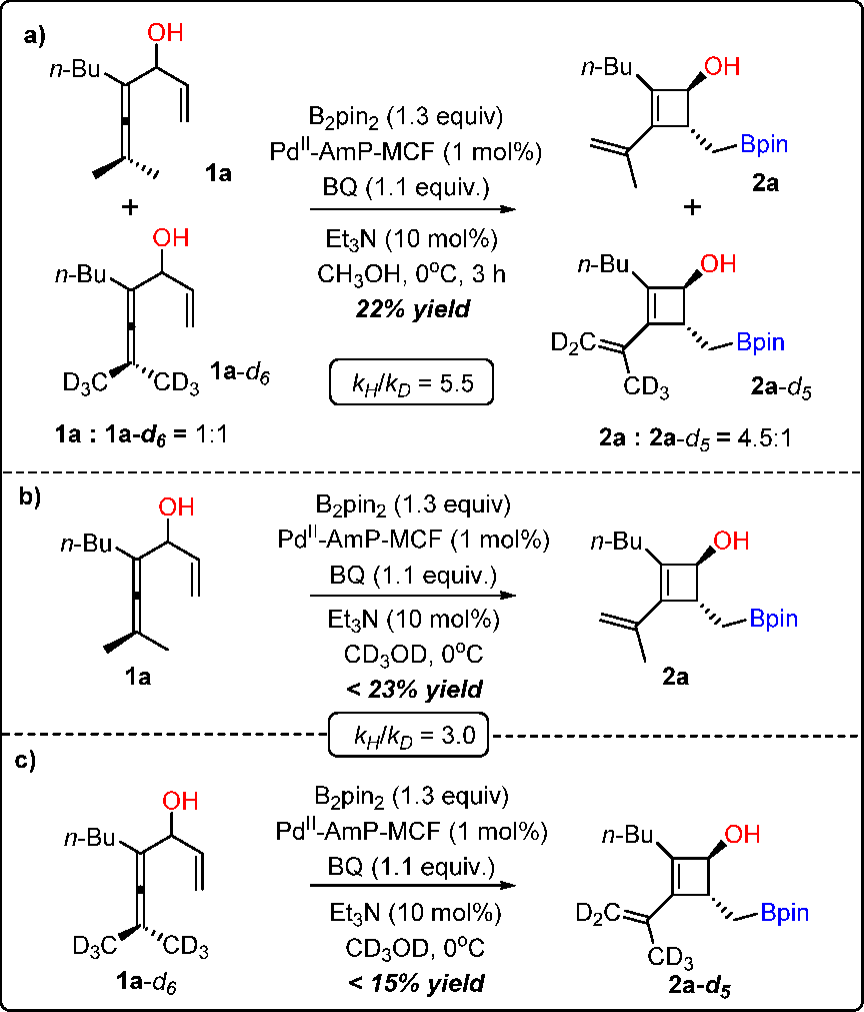
KIE Studies of This Heterogeneous Process

Deuterium kinetic isotope effect (KIE) studies indicate that C–H bond cleavage (from *Int*-**1** to *Int*-**2** in Scheme 2c) is the rate-limiting and first irreversible step in this heterogeneous process (Scheme 3). To rule out the possibility that the leached Pd species catalyze the reaction, we conducted inductively coupled plasma optical emission spectroscopy (ICP-OES, detection limit: 0.02 μg/mL) analysis of the liquid phase taken from the reaction mixture. The result showed that there was no detectable leaching of Pd in the reaction solution (<0.1 ppm). Hot filtration tests also confirmed this conclusion, which suggests a heterogeneous pathway.^39^

**Scheme 4:**
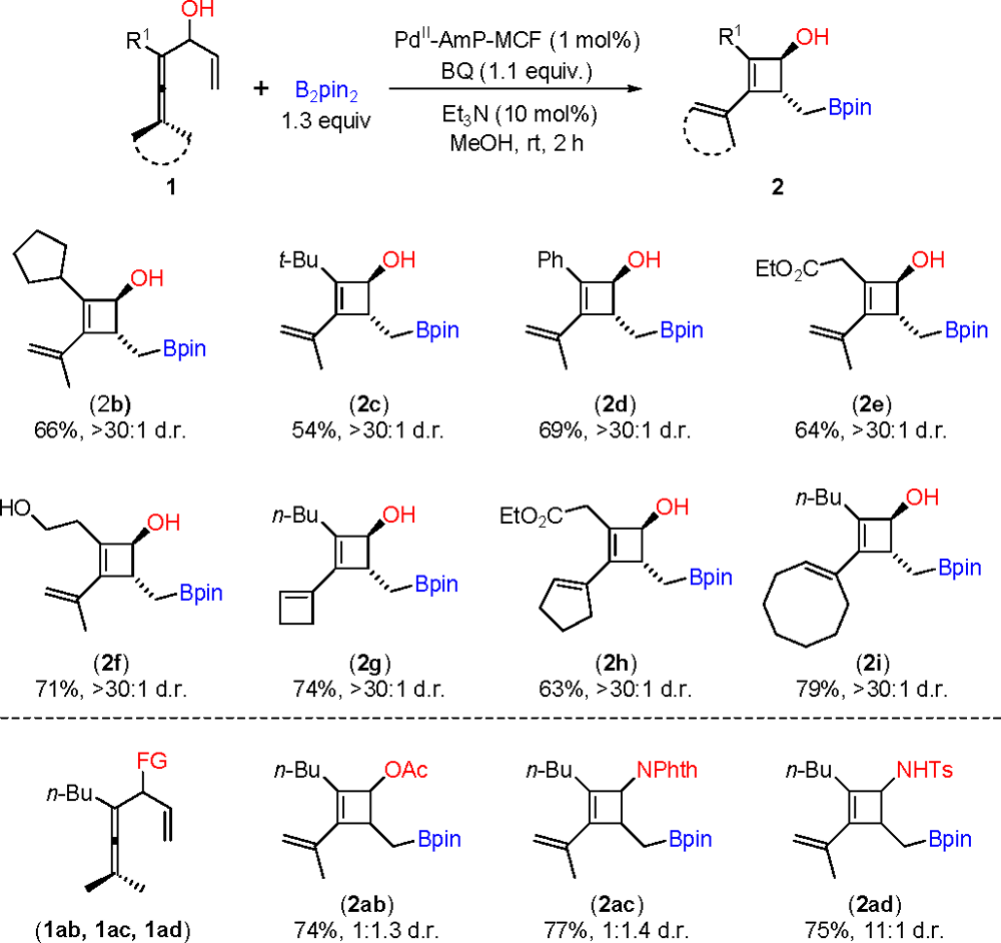
Oxidative Carbocyclization-Borylation Towards Cyclobutenols with High Diastereoselectivity

Cyclobutenol is a versatile building block and key element in a large number of bioactive compounds and natural products.^40a^ The most straightforward route to cyclobutene derivatives via a [2 + 2]-cycloaddition of an alkyne and an olefin cannot be used for the construction of this skeleton.^40b^ Moreover, the diastereoselectivity control as seen in the current method for preparing cyclobutenol is challenging.^41^ Under the optimized reaction conditions, enallenols **1** reacted with B_2_pin_2_, furnishing *trans*-cyclobutenols **2** with good substrate tolerance and high diastereoselectivity (Scheme 4). This heterogeneous method is a useful supplement to the synthetic route of cyclobutenols. It is noteworthy that an active hydrogen (OH or NH) is required in the substrate for controlling the diastereoselectivity, and the replacement of OH or NHTs by an acetyl group (OAc) or phthalimide group (NPhth) resulted in poor selectivity.

This initial try^38^ gave us a positive indication that Pd-AmP-MCF with BQ is catalytically active in an oxidative cascade reaction of an allene via a heterogeneous pathway. Inspired by this work, in the following 2 years, we developed a series of heterogeneous palladium-catalyzed oxidative cascade processes toward the construction of complex compounds with high efficiency and selectivity.

## 3 High Palladium Efficiency and Solvent-Controlled Chemoselectivity of the Heterogeneous Palladium Catalysts

### 3.1 High Palladium Efficiency

**Scheme 5:**
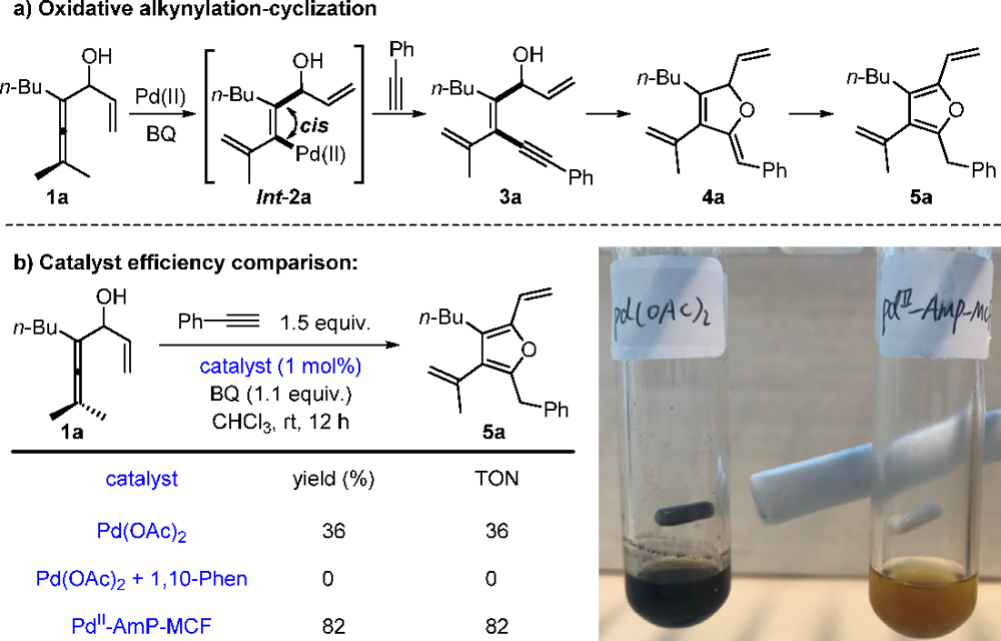
Catalyst Efficiency Comparison in Oxidative Alkynylation-Cyclization toward Furans

It is generally considered that heterogeneous palladium catalysts are less active and selective than their homogeneous counterparts as a result of the mass-transfer issue.^42^ In our studies, we found the heterogeneous Pd^II^-AmP-MCF to be highly efficient in oxidative cascade reactions of allenes. In the oxidative alkynylation-cyclization reaction of enallenols with alkynes for the synthesis of furans (Scheme 5), the yields and TONs of Pd(OAc)_2_ and Pd^II^-AmP-MCF are shown for comparison.^2^ The use of 1 mol % of Pd(OAc)_2_ led to a catalytic reaction, giving only 36% yield of furan **5a** with over half of the starting material **1a** being recovered, while Pd^II^-AmP-MCF with the same Pd loading (1 mol %) gave an 82% yield of **5a**. It is noteworthy that the addition of a bidentate N-ligand to the homogeneous Pd(OAc)_2_ results in catalyst passivation, probably due to the strong coordination disfavoring the formation of *Int***-2a** (Scheme 5a).

**Figure 3:**
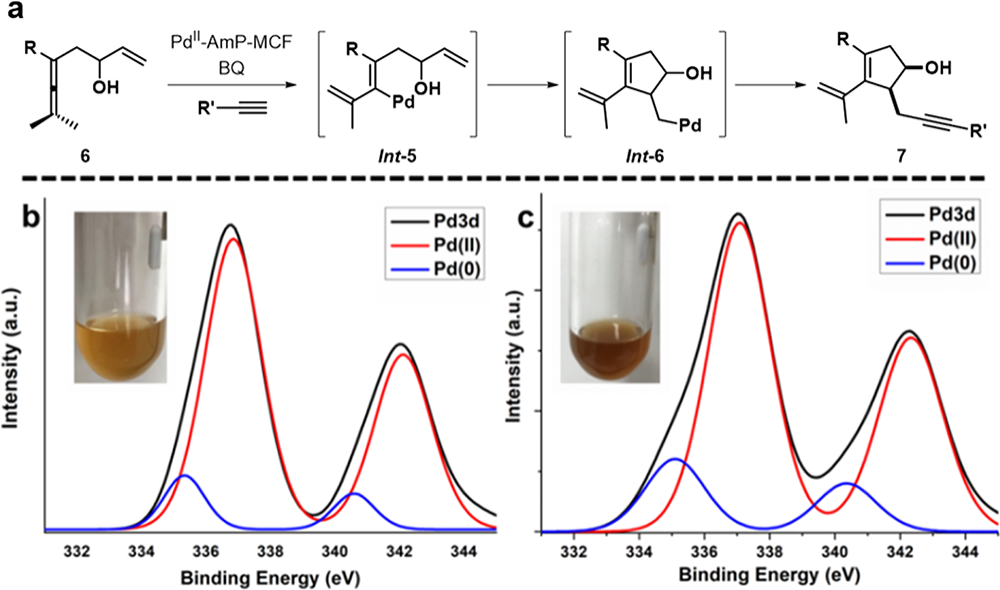
Pd^II^-AmP-MCF-catalyzed oxidative carbocyclization-alkynylation (a) and the XPS analysis of the heterogeneous catalyst before (b) and after (c) reaction

As shown in Scheme 5b, it is clear that a considerable amount of Pd black was generated from aggregation under the homogeneous reaction conditions, while this aggregation was not observed under the heterogeneous conditions with Pd^II^-AmP-MCF.^2^ We conclude that the porous amino-functionalized support (AmP-MCF) protects the palladium species from aggregation, thus circumventing the Pd deactivation problem. This conclusion was further confirmed by XPS analysis of the heterogeneous catalyst in the oxidative carbocyclization-alkynylation reaction by using enallenol **6** as the starting material (Figure 3a): XPS spectra of Pd^II^-AmP-MCF before and after reaction showed a similar Pd(II) dominance (Figure 3b and c).^4^

**Figure 4:**
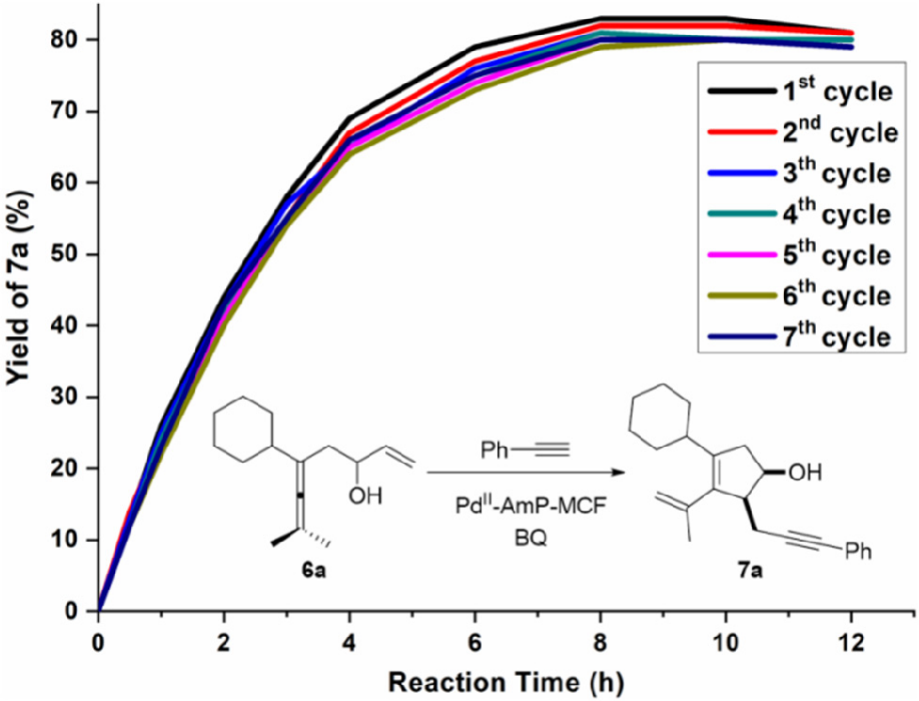
Recycling experiments and kinetic studies.

Pd^II^-AmP-MCF exhibits high palladium efficiency, on the one hand owing to its high TONs, and on the other hand due to its recyclability. It is recoverable and recyclable in the oxidative cascade reactions. Additionally, kinetic studies by using enallenol **6** as the starting material revealed that not only the yield, but also the reaction rate was essentially maintained between the first and the seventh runs,^4^ which demonstrates that Pd^II^-AmP-MCF is robust and highly active under the heterogeneous reaction conditions (Figure 4). It should be pointed out that although the heterogeneous catalyst shows good recyclability during seven cycles, we cannot exclude the possibility that it might slowly lose the catalytic activity after a large number of runs.^43^

### 3.2 Solvent-Controlled Chemoselectivity

**Scheme 6:**
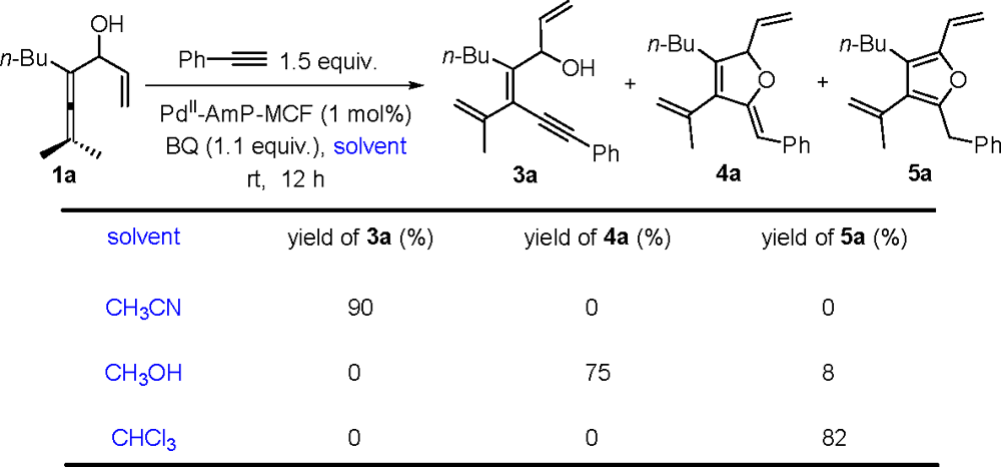
Solvent-Controlled Selectivity in Oxidative Alkynylation-Cyclization

**Scheme 7:**
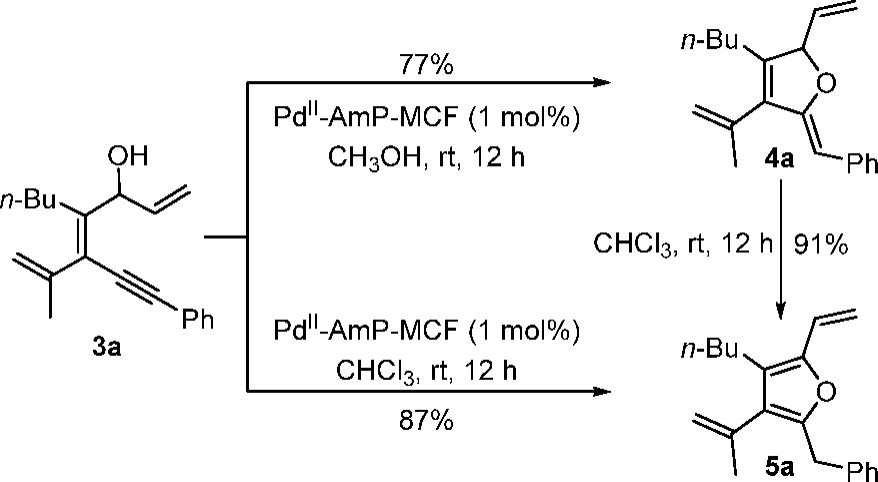
Control Experiments on the Transformation from **3** to **4** and then **5**

Interestingly, the Pd^II^-AmP-MCF-catalyzed oxidative cascade reactions of enallenols (**1** or **6**) involving terminal alkynes as the reaction partner exhibited a solvent-controlled chemoselectivity. In the reaction of enallenol **1a** with alkyne,^2^ (*Z*)-tetrasubstituted olefin **3a**, 2,5-dihydrofuran **4a**, or tetrasubstituted furan **5a** was isolated as the major product by using CH_3_CN, CH_3_OH, or CHCl_3_, respectively, as the solvent (Scheme 6). We speculated that **4** and **5** were generated from the Pd(II)-catalyzed intramolecular cyclization of **3** and isomerization of **4**, respectively, which was confirmed by the further control experiments (Scheme 7). The solvent-controlled selectivity could be explained by (a) the interaction of CH_3_CN with Pd(II) inhibiting the subsequent Pd-catalyzed cyclization of **3**, (b) the protic solvent (CH_3_OH) promoting transformation of **3** to **4**, as a proton is essential during the process, and (c) the relatively acidic reaction conditions with the use of CHCl_3_ (2CHCl_3_ + O_2_ → 2COCl_2_ + 2HCl) favoring the isomerization from **4** to **5**.^2^

**Scheme 8:**
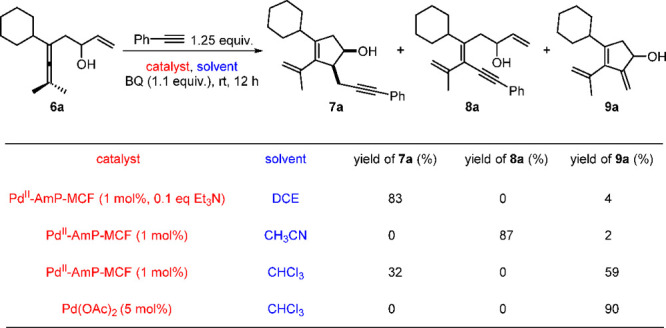
Solvent/Catalyst-Controlled Chemoselectivity in Oxidative Carbocyclization-Alkynylation

**Scheme 9:**
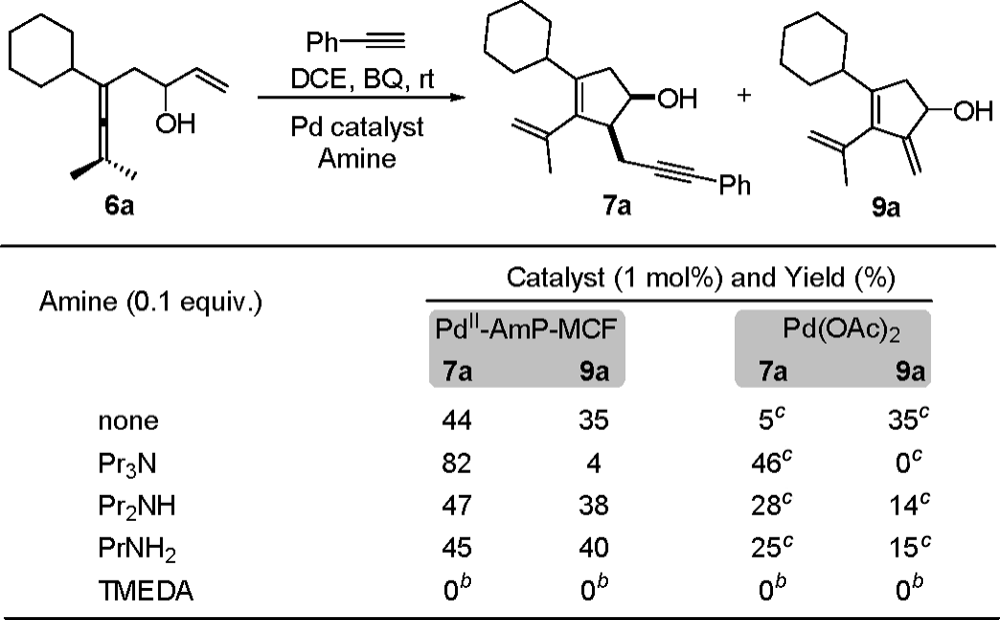
Control Experiments on the Use of Different Amine Additives Reaction conditions: **6a** (0.2 mmol), phenylacetylene (0.25 mmol), Pd catalyst (1 mol%), amine additive (0.1 equiv.), BQ (1.1 equiv), DCE (1.0 mL), 8 h. **6a** was recovered in >90% yield. **6a** was partially recovered.

In the reaction of enallenol **6a** with alkyne,^4^ cyclopentenol **7a**, (*E*)-tetrasubstituted olefin **8a**, or **9a** was the major product by using 1,2-dichloroethane (DCE), CH_3_CN, or CHCl_3_, respectively, as the solvent under the heterogeneous reaction conditions (Scheme 8). However, in CHCl_3_, there was still 32% yield of **7a** in addition to the major product **9a**. Based on the result that the addition of catalytic amounts of Et_3_N further improved the selectivity of the oxidative carbocyclization-alkynylation reaction to give **7a** as the dominant product, we speculated that apart from the solvent, the amine additive also played an important role in altering the chemoselectivity of the reaction. Control experiments on using different amine additives confirmed our speculation (Scheme 9). These results demonstrate that the coordination of amine to Pd(II) improves the selectivity to give **7a**, probably via suppressing the β-H elimination or promoting the alkyne ligand exchange of *Int***-6** (Figure 3a). This could explain the result that 32% yield of **7a** was still isolated in CHCl_3_ by using Pd^II^-AmP-MCF as the catalyst, considering that the Pd(II) in Pd^II^-AmP-MCF is coordinated by amine (NH_2_). Based on this conclusion, Pd(OAc)_2_ was used in place of Pd^II^-AmP-MCF in CHCl_3_, and the selectivity of the reaction was completely switched to give **9a** as the exclusive product in 90% yield under the homogeneous reaction conditions (Scheme 8). It is noteworthy that 5 mol % of Pd(OAc)_2_ is required for full conversion of the starting material **6a**, which demonstrates that Pd^II^-AmP-MCF is more efficient than the homogeneous palladium catalyst (Pd(OAc)_2_).

**Scheme 10:**
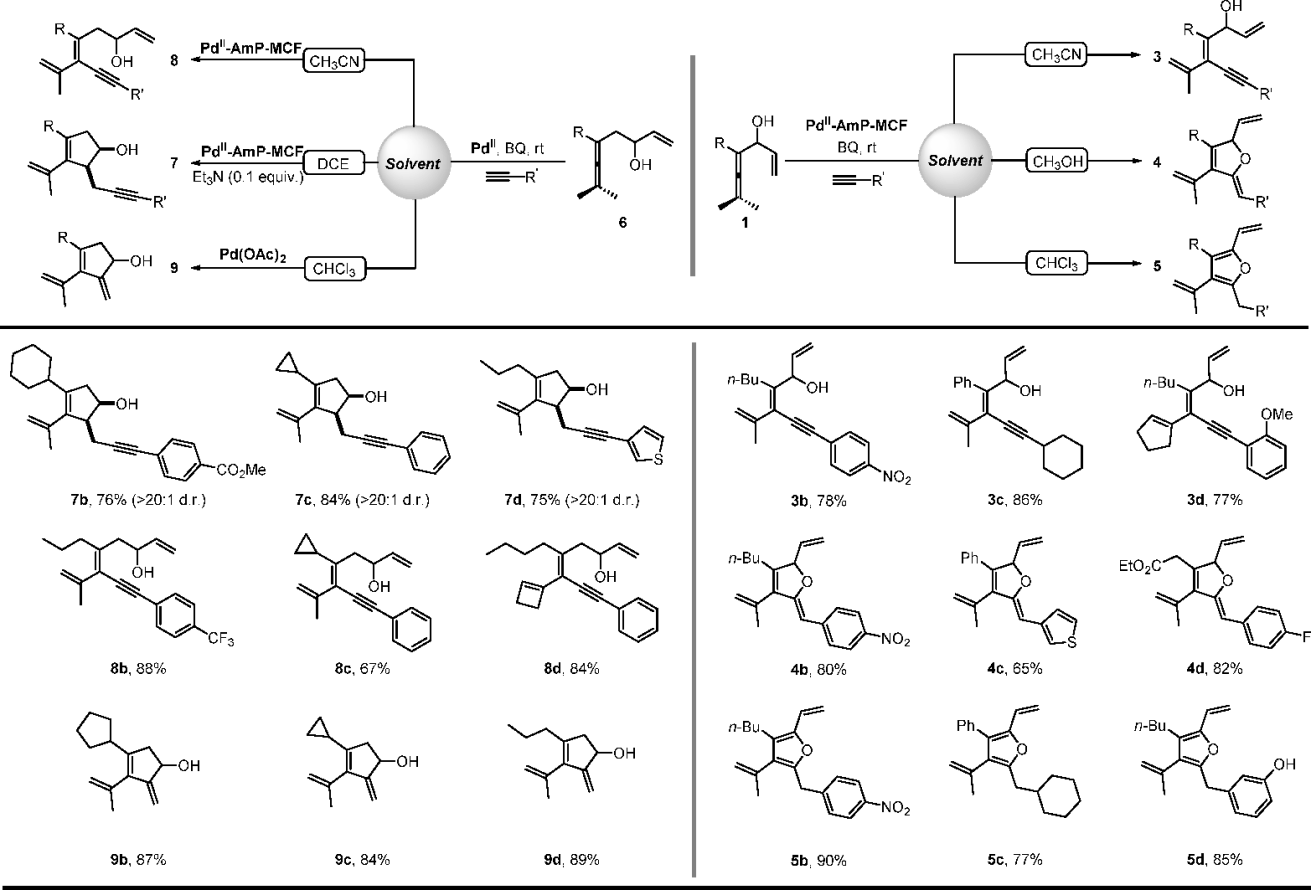
Solvent-Controlled Chemodivergent Syntheses

The solvent-controlled selectivity was utilized for the chemodivergent synthesis of highly substituted olefins, furans, and cyclopentenols (Scheme 10). Notably, the heterogeneous palladium-catalyzed oxidative cascade reactions not only displayed good chemoselectivity, but also showed excellent stereoselectivity. Cyclopentenols **7** were isolated as single *cis*-diastereomers in high yield in each case, and the olefins **3**, **4**, and **8** were obtained with stereodefined double bonds under the reaction conditions.^2,4^

## 4 Unique Selectivity of the Heterogeneous Palladium Catalysts

**Scheme 11:**
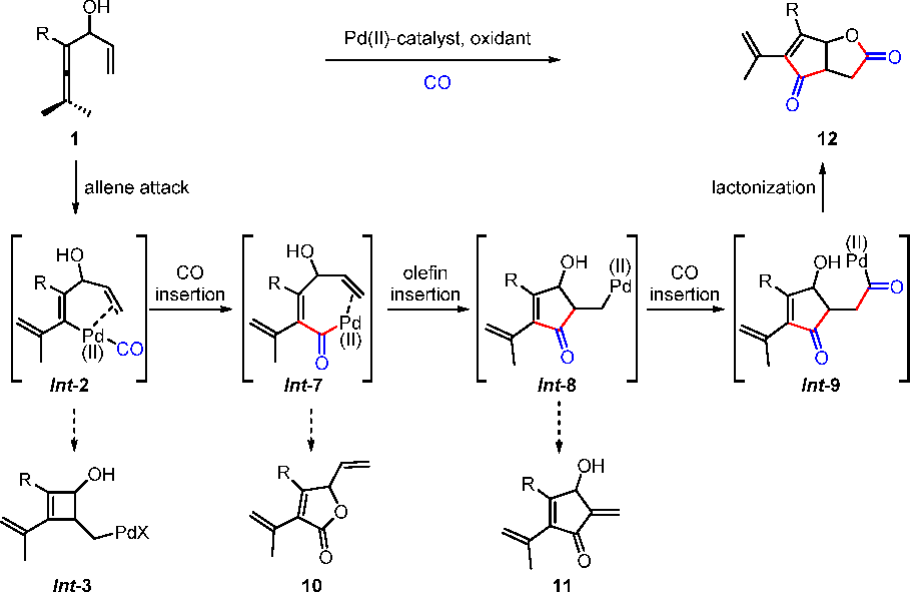
Oxidative Carbocyclization-Carbonylation for the Construction of Fused γ-Lactone

Pd(II)-catalyzed oxidative carbonylation constitutes an efficient approach toward synthesis of organic molecules with carbonyl groups.^32,44,45^ We designed an oxidative cascade route involving carbonylation^1,46^ for the construction of ring fused γ-lactone **12**, which is a core structure of many bioactive compounds, such as the strigolactone family members.^47^ The envisioned cascade process (Scheme 11) for the construction of this skeleton involves four steps of bond formation including carbon monoxide (CO) insertions twice in the reaction sequence.^1^ The possible side reactions of each palladium intermediate result in difficulties of controlling the chemoselectivity of the cascade reaction.

**Scheme 12:**
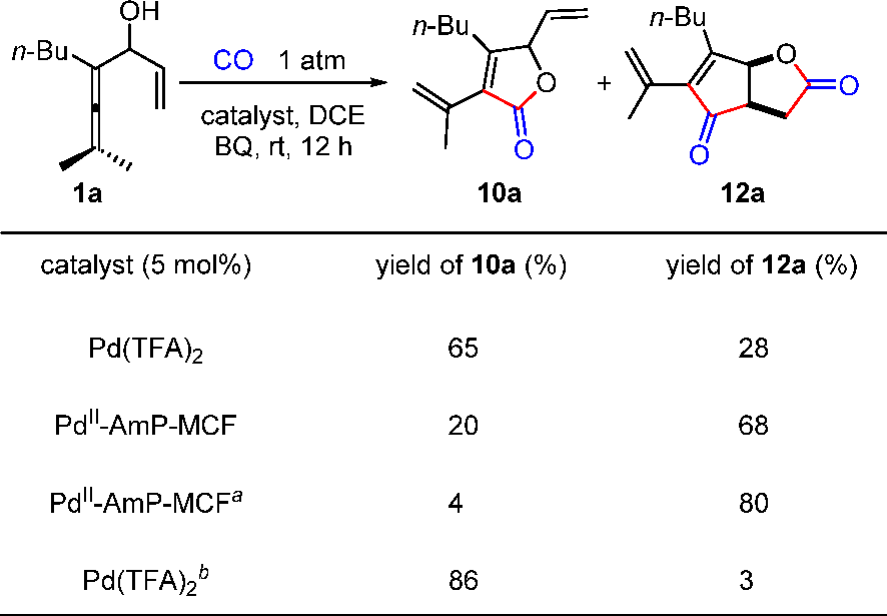
Comparison of the Selectivity by Using Homogeneous/Heterogeneous Catalyst Pd^II^-AmP-MCF (2 mol% ), methyl-BQ. 10 mol% of AcOH was used.

**Scheme 13:**
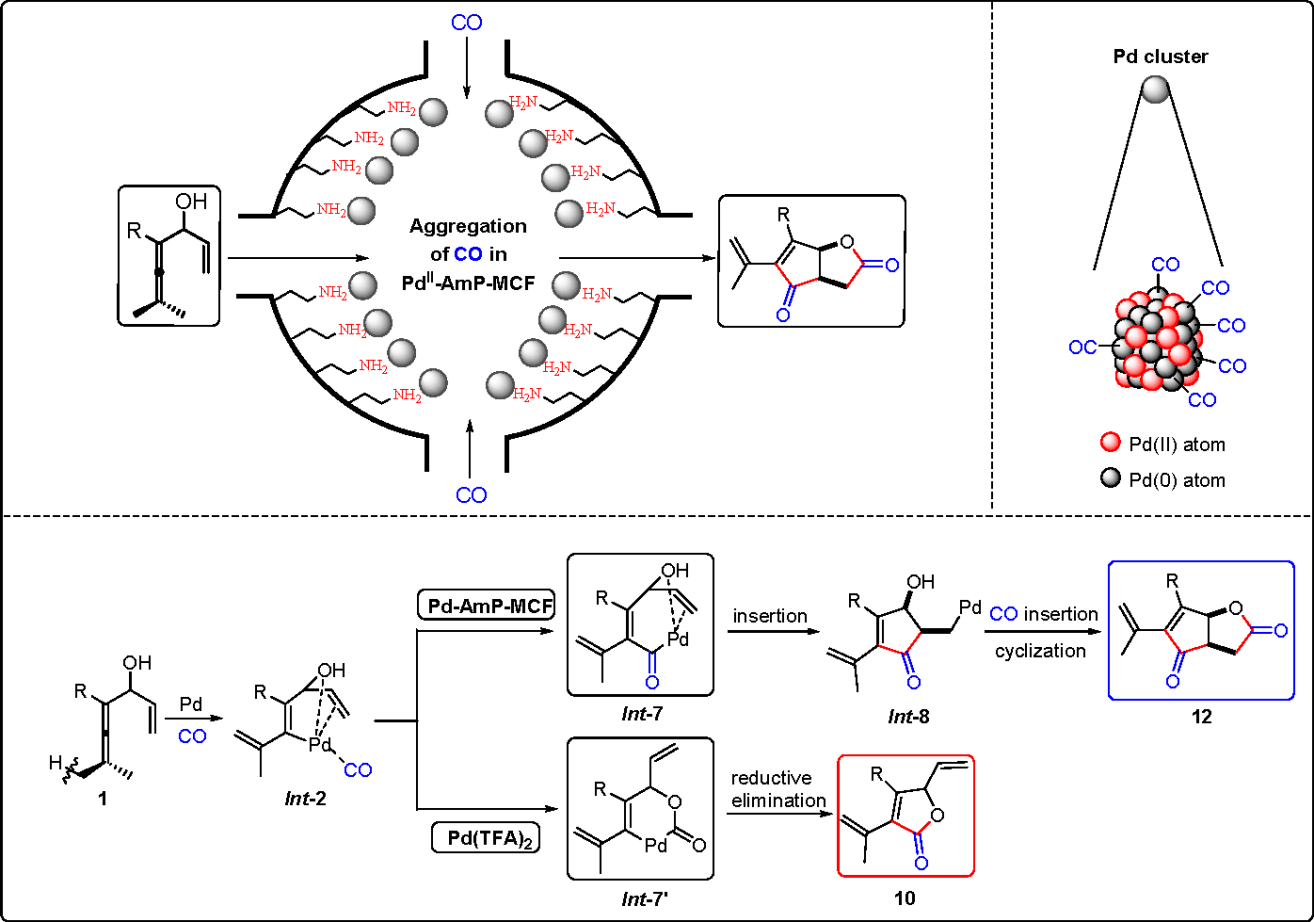
Origin of the Selectivity

Initial attempts to use **1a** in the envisioned oxidative cascade reaction resulted in **10a** as the major product under all homogeneous reaction conditions tried. Typically, in the presence of Pd(TFA)_2_, **10a** and **12a** were obtained in 65% and 28% yield, respectively (Scheme 12). However, when Pd^II^-AmP-MCF was used in place of the homogeneous palladium catalyst, the selectivity of the reaction was completely reversed, offering **12a** in 68% yield. A slight modification of the oxidant under the heterogeneous reaction conditions improved the yield of **12a** to 80%. The homogeneous Pd(TFA)_2_-catalyzed reaction, with 10 mol % of AcOH as an additive, gave **10a** in 86% yield. The results of control experiments^1^ suggest that the distinct chemoselectivity between Pd(TFA)_2_ and Pd^II^-AmP-MCF originated from their different adsorption (coordination) capacity for CO. The high surface area of Pd^II^-AmP-MCF and presence of Pd(0) atoms favor adsorption and coordination of CO in the catalyst, which results in the aggregation of CO in Pd^II^-AmP-MCF. The local high CO concentration would promote the generation of *Int***-7** from *Int***-2** with subsequent carbocyclization to give *Int***-8**. A second CO insertion of *Int***-8** would give **12**. On the other hand, a low CO concentration would favor the formation of *Int***-7′** from *Int***-2** with subsequent reductive elimination to give **10**. (Scheme 13).

**Scheme 14:**
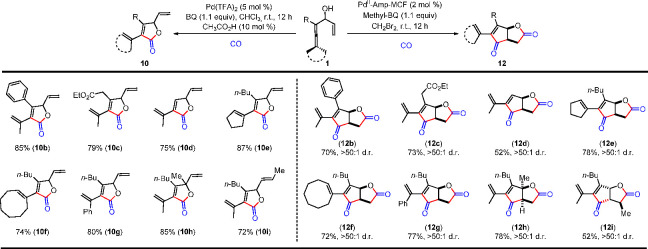
Chemodivergent Syntheses of γ-Lactone

**Scheme 15:**
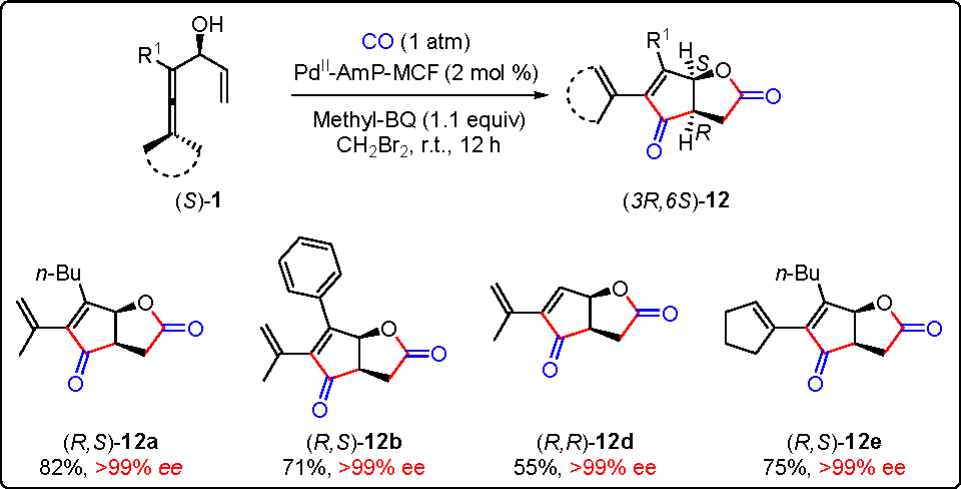
Synthesis of Enantiomerically Pure Fused γ-Lactones

The unique selectivity of Pd^II^-AmP-MCF-catalyzed oxidative carbocyclization-carbonylation reaction allows chemodivergent construction of γ-lactone **10** and fused γ-lactone **12**.^1^ It is noteworthy that **12** bearing two chiral centers were obtained with high diastereoselectivity as the *cis*-product (Scheme 14). Synthesis of optically pure fused γ-lactones **12** was realized by the use of (*S*)-enallenols **1** as starting materials in the stereoselective cascade reaction (Scheme 15). (*S*)-Enallenols **1** were readily obtained from kinetic resolution of enallenols **1** with *C. antarctica* lipase B (CalB).

## 5 Silver-Triggered High Activity of the Heterogeneous Palladium Catalysts

**Scheme 16:**
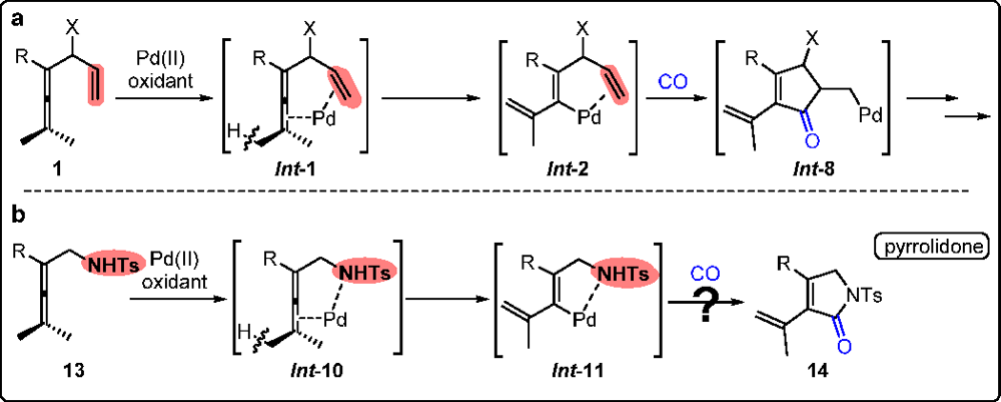
Oxidative Carbonylation-Cyclization Process for the Construction of Pyrrolidone

**Figure 5:**
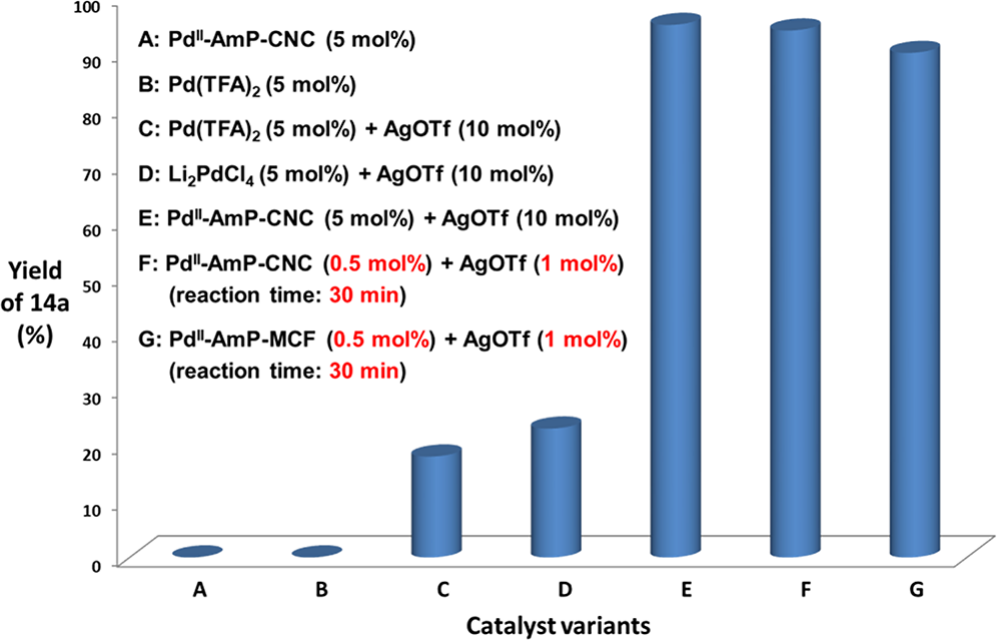
Catalyst efficiency comparison of palladium-catalyzed oxidative carbonylation-cyclization.

Based on our previous reports, an unsaturated carbon–carbon bond is essential to trigger the allenic attack via coordination to Pd(II) in oxidative carbonylation reactions (Scheme 16a).^31,32,48^ In 2019, we found that the sulfonamide group of α-tosylamide allenes could undergo anionic ligand exchange with ligands on the Pd(II) catalyst. The coordination of the sulfonamide group also triggered the allenic attack step and the subsequent alkynylation.^49^ Inspired by this observation, we designed an oxidative carbonylation-cyclization process for the construction of pyrrolidones (Scheme 16b).^3^ Initial attempts, however, gave very low yield of pyrrolidone **14a** (<8%), and variation of solvent, oxidant, or temperature did not improve the yield with the starting material **13a** being almost completely recovered. After screening different additives, finally we found that the addition of catalytic amounts of AgOTf (10 mol %) in Pd^II^-AmP-MCF dramatically improved the yield of **14a** to >90% (Figure 5). Further optimization of the reaction conditions showed that the loading of Pd^II^-AmP-MCF and AgOTf could be reduced to 0.5 mol % and 1 mol %, respectively, furnishing **14a** in satisfactory yield in 30 min. By using crystalline nanocellulose as the support, we prepared Pd^II^-AmP-CNC as a catalyst by using the same procedure as the synthetic method of Pd^II^-AmP-MCF (Figure 1a). Pd^II^-AmP-CNC also showed high catalytic activity with the addition of AgOTf in this oxidative cascade reaction. In contrast, homogeneous palladium catalysts such as Pd(TFA)_2_ or Li_2_PdCl_4_ did not give satisfactory yields with AgOTf (Figure 5).

**Figure 6:**
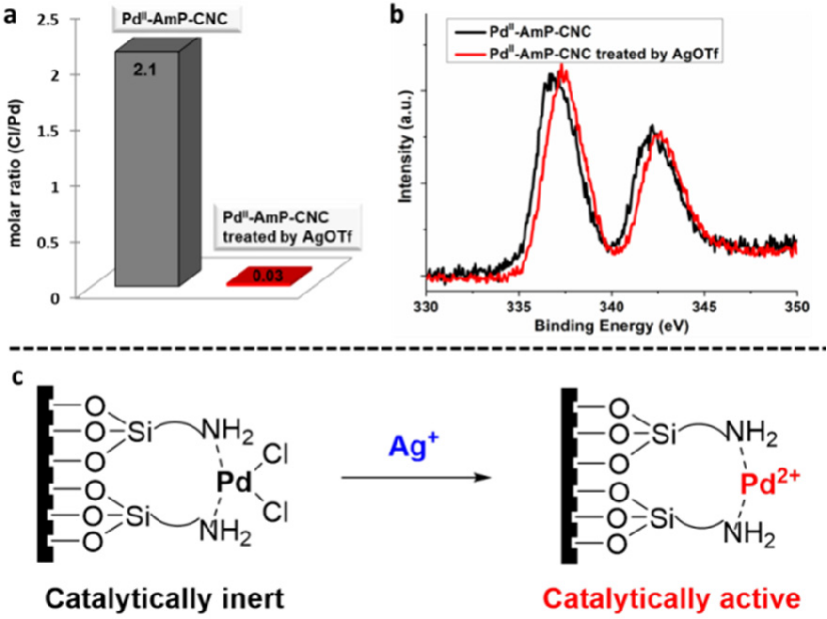
Identification of active palladium species.

To reveal the origin of the high activity of this catalytic system (Pd^II^-AmP-CNC + AgOTf) in the oxidative carbonylation-cyclization reaction, we conducted control experiments.^3^ The results show that the Cl/Pd molar ratio of Pd^II^-AmP-CNC is 2.1/1 and 0.03/1 before and after treatment with AgOTf, respectively (Figure 6a). The analysis of XPS spectra indicates that the Pd 3d binding energy of Pd^II^-AmP-CNC treated by AgOTf is higher than that of untreated catalyst (Figure 6b). These results suggest that the chloride on the palladium clusters of Pd^II^-AmP-CNC has been completely removed by AgOTf, generating cationic palladium^50^ with high activity in the oxidative carbonylation-cyclization reaction (Figure 6c). The addition of AgOTf to homogeneous palladium also results in an improvement of production of **14a** (Figure 5), which supports the generation of cationic palladium. However, the homogeneous catalytic system did not give satisfactory yield, probably due to the immediate deactivation of the palladium species under the reaction conditions.

**Scheme 17:**
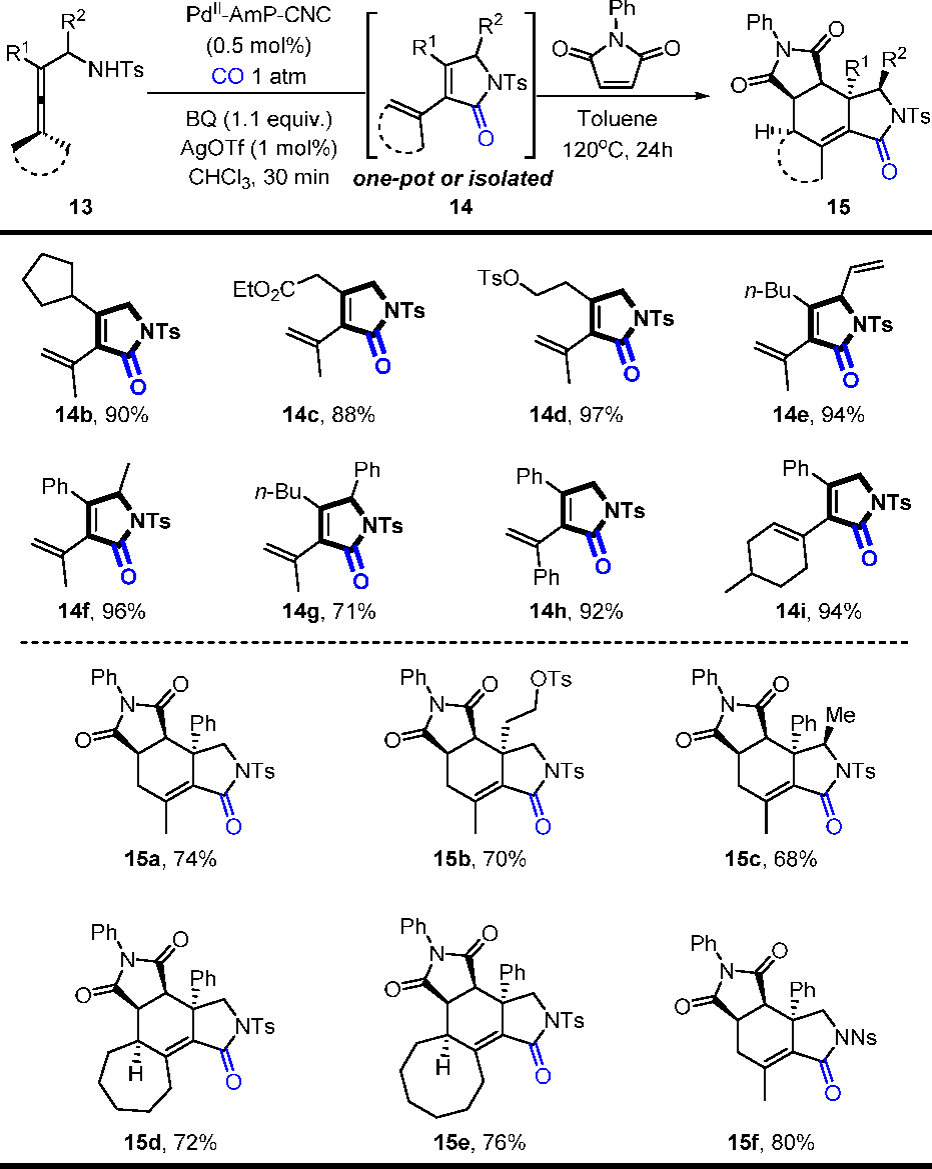
Rapid Access to Pyrrolidones and Highly Diastereoselective Synthesis of Polycycles

This heterogeneous method allows rapid access to pyrrolidones with high efficiency, and it has been utilized for the one-pot construction of polycyclic compounds with a subsequent Diels–Alder reaction (Scheme 17). It is noteworthy that polycyclic compounds **15** were obtained with high diastereoselectivity and only 0.5 mol % Pd^II^-AmP-CNC catalyst and 1 mol % AgOTf were needed in the oxidative cascade reaction.

## 6 Outlook

**Figure 7:**
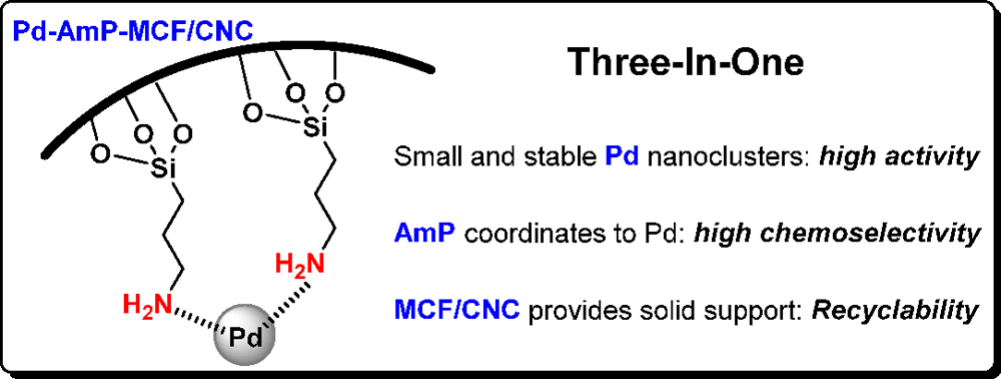
Three-In-One role of Pd-AmP-MCF/CNC in oxidative cascade reactions.

In the past three years, we have developed a series of oxidative cascade reactions based on the efficient heterogeneous palladium catalyst Pd-AmP-MCF/Pd-AmP-CNC.^1−4,38,46^ This kind of catalyst consists of a solid support (MCF or CNC), amino linkers (AmP), and palladium clusters (Pd). The palladium catalyst is oxidized by BQ to generate an active Pd species, which is highly active in catalyzing oxidative cascade reactions of allene derivatives. The amino linkers are able to adjust the selectivity of the reaction. Meanwhile, this solid support allows efficient recovery and recycling of the heterogeneous palladium catalyst. In other words, this catalyst plays a Three-In-One role in oxidative cascade reactions, exhibiting high activity and selectivity as well as recyclability (Figure 7). Many useful compounds such as cyclobutenols, furans, ring fused γ-lactones, and pyrrolidones have been synthesized with high chemo- and diastereoselectivity by this heterogeneous method. Silver-triggered high activity of Pd-AmP-MCF (and Pd-AmP-CNC) has been explored very recently. More work on the use of this efficient heterogeneous palladium catalyst for oxidative cascade reactions is under way in our laboratory.

Despite some successful examples on heterogeneous palladium-catalyzed oxidations, homogeneous palladium catalysis is still dominant in this research field. To tap the potential of heterogeneous palladium catalysis in oxidative processes, rational design of efficient catalysts is of great importance and the following three proposed approaches could significantly stimulate further development of the field: (1) Robust heterogeneous nanopalladium catalysts with ultrasmall particle size would be able to overcome the mass-transfer issues and dramatically increase the catalytic activity. (2) Metal-doping in Pd nanoclusters would lead to a novel heterogeneous palladium catalysts, where the new metal introduced (e.g., Cu, Au) would cooperate with palladium and synergistically catalyze oxidative cascade reactions, realizing organic transformations that are hard to achieve by mononuclear homogeneous palladium catalysts. (3) Design and synthesis of well-defined palladium nanoclusters with atomically precise structures,^51^ and their applications in catalytic oxidations, will be helpful for in-depth understanding of structure–property correlations and catalytic mechanisms, promoting the recursive optimization of structure and catalytic performance of the heterogeneous palladium catalyst.
